# *Escherichia coli-*derived virus-like particles in vaccine development

**DOI:** 10.1038/s41541-017-0006-8

**Published:** 2017-02-09

**Authors:** Xiaofen Huang, Xin Wang, Jun Zhang, Ningshao Xia, Qinjian Zhao

**Affiliations:** 1 State Key Laboratory of Molecular Vaccinology and Molecular Diagnostics, National Institute of Diagnostics and Vaccine Development in Infectious Diseases, Xiamen University, Xiamen, Fujian 361102 PR China; 2 School of Public Health, Xiamen University, Xiamen, Fujian 361102 PR China; 3 School of Life Science, Xiamen University, Xiamen, Fujian 361102 PR China

## Abstract

Recombinant virus-like particle-based vaccines are composed of viral structural proteins and mimic authentic native viruses but are devoid of viral genetic materials. They are the active components in highly safe and effective vaccines for the prevention of infectious diseases. Several expression systems have been used for virus-like particle production, ranging from *Escherichia coli* to mammalian cell lines. The prokaryotic expression system, especially *Escherichia coli*, is the preferred expression host for producing vaccines for global use. Hecolin, the first licensed virus-like particle vaccine derived from *Escherichia coli*, has been demonstrated to possess good safety and high efficacy. In this review, we focus on *Escherichia coli*-derived virus-like particle based vaccines and vaccine candidates that are used for prevention (immunization against microbial pathogens) or disease treatment (directed against cancer or non-infectious diseases). The native-like spatial or higher-order structure is essential for the function of virus-like particles. Thus, the tool box for analyzing the key physicochemical, biochemical and functional attributes of purified virus-like particles will also be discussed. In summary, the *Escherichia coli* expression system has great potentials for producing a range of proteins with self-assembling properties to be used as vaccine antigens given the proper epitopes were preserved when compared to those in the native pathogens or disease-related target molecules.

## Introduction

Vaccination is the most efficient way to control and prevent infectious diseases. Currently, the majority of licensed vaccines produced by traditional technologies are either live-attenuated or inactivated, although both may present safety issues (such as reversion to virulence and residual virulence).^1^ In the 1970s, scientists discovered that a single key protein from a virus could be a vaccine antigen.^2, 3^ Almost a decade later, the first genetically engineered vaccine using recombinant gene expression technology was produced in the prokaryotic microbe.^4^ With the advent of modern molecular biology, recombinant subunit vaccines have flourished in human vaccinology. Virus-like particles (VLPs) are composed of the virion-building proteins of a virus and spontaneously self-assemble into particles without incorporating the infective viral genome.^5^ Thus, VLP are extremely promising vaccine candidates due to their native-like and non-infective properties. VLPs can induce both innate and adaptive immune responses and have shown to be highly immunogenic in animals and humans.^5, 6^ The approved VLP-based vaccines have been produced in yeast,^7, 8^ insect, bacteria *Escherichia coli* (*E. coli*),^9, 10^ plant, and mammalian cells,^11, 12^ ranging from prokaryotic to eukaryotic expression systems.

**Fig. 1:**
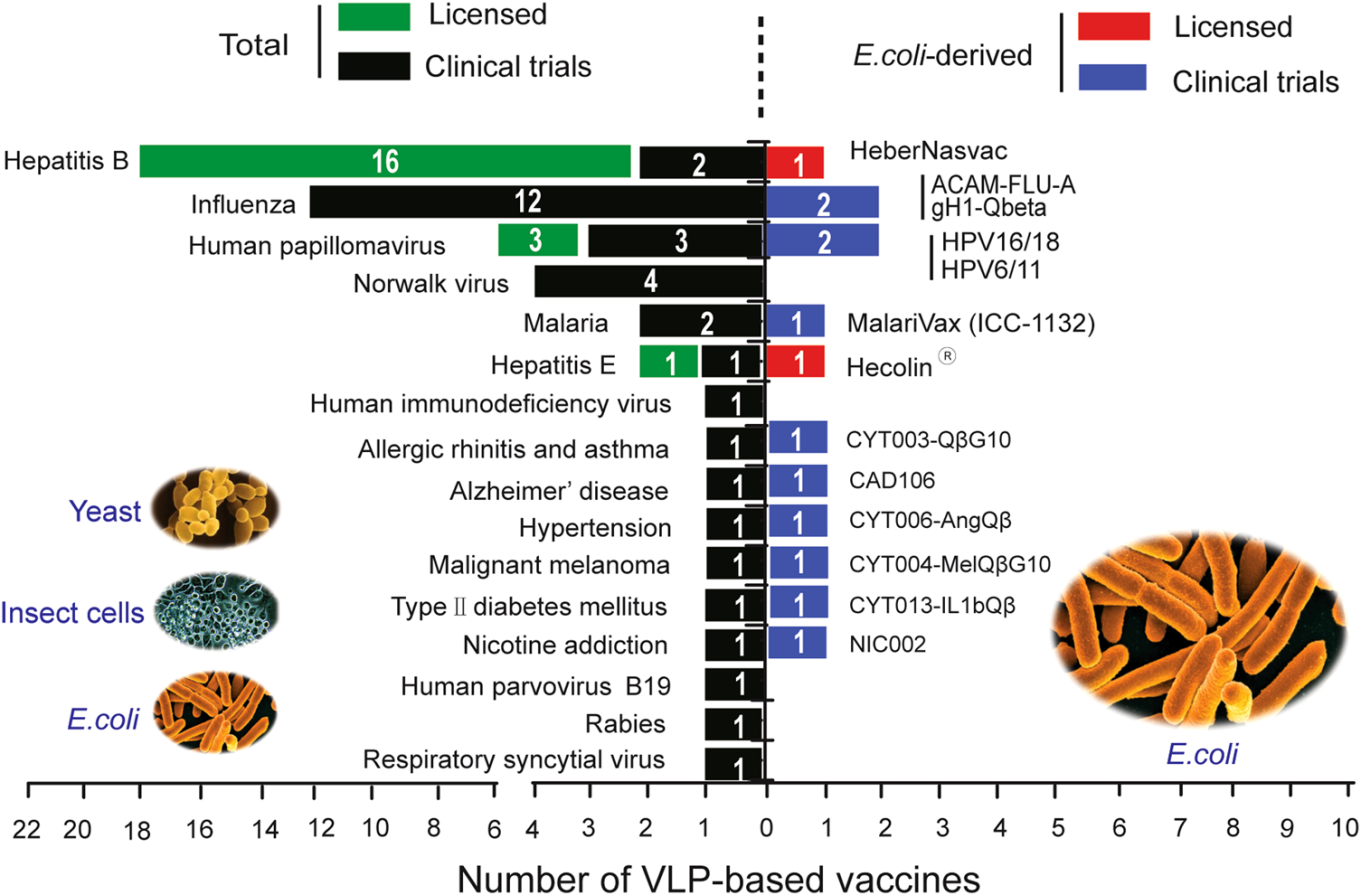
The number of VLP-based vaccines or vaccine candidates that were approved and in clinical studies from 1986 to 2015. Total numbers of VLP vaccines or candidates derived from different expression systems (including *E. coli*) are plotted on the left. The numbers of commercialized VLP vaccines or those being tested in clinical trials derived from *E. coli* are plotted on the right.

Bacteria, especially *E. coli*, have been widely used for producing recombinant proteins. The first recombinant pharmaceutical approved for the treatment of diabetes, recombinant human insulin (Humulin-US/Humuline-EU), was obtained from *E. coli*.^13^ Numerous recombinant protein-based products derived from *E. coli* have been approved for therapeutic use, including cytokines, hormones, growth factors, serine proteases, and fusion proteins.^14^ The food and drug administration and European medicines agency have approved 151 protein-based recombinant drugs, 45 (29.8%) of which are produced using products derived from *E. coli*.^15^ From 2010 to July 2014, almost 33% of the approved recombinant biopharmaceutical in the United States and EU were obtained from Chinese hamster ovary cells, while 29% and 16.5% were obtained from *E. coli* and yeast, respectively.^16^ Thus, *E. coli* is still a widely used host for the production of protein-based biopharmaceuticals. Of the 174 different types of VLPs successfully produced, approximately 28% were produced in bacterial systems, 20% in yeast systems and 28% in insect systems.^17^ As of 2015, over 50 VLP-based vaccines or vaccine candidates, derived from different expression hosts, have been licensed or are under clinical development (Fig. 1, Supplementary Table 1S). Hecolin was the first VLP-based vaccine against hepatitis E virus (HEV) obtained from *E. coli* and was licensed by the FDA of China, or CFDA, in 2011.^18, 19^ Currently, scientists in academia and industry are actively seeking ways to produce more cost-effective VLP-based vaccines, particularly low-cost vaccines for distribution in developing countries.

VLP-based vaccines are pre-eminent candidates for vaccination because of their high immunogenicity and good safety performance.^5, 20^ VLP-based vaccines derived from *E. coli* are more cost-effective than those derived from insect cells or yeasts during industrial production. However, only one VLP-based vaccine, Hecolin, fully derived from *E. coli* has been approved for use in humans.^20^ Currently, a number of recombinant specific *E. coli* strains have been developed with the intention of achieving high-yield and high-quality protein production. Proper folding that allows the formation of specific structures is essential for the function of VLPs in clinical use. Various in vitro analytical methods, in conjunction with in vivo evaluation, have been established to monitor the presence of native-like epitopes on VLPs obtained from *E. coli*, thereby ensuring the efficacy of VLP-based vaccines. This review highlights examples of *E. coli*-derived VLP-based vaccines and vaccine candidates. Additionally, the functional assessment of VLPs and the challenges associated with recombinant VLP proteins produced in *E. coli* will also be discussed.

## Why use *E. coli*-derived VLPs for vaccine development?

As a robust protein expression host, *E. coli* has many advantages, such as inexpensive culturing, high expression levels, easy scale-up and short turnaround time.^21, 22^ It is a preferred expression system for protein production if the protein can be correctly folded. In vaccine development, high product cost would restrict utilization, especially in developing countries.^23^ Existing, emerging and re-emerging infectious diseases pose a threat to human life and productivity in both the developed and the developing world. There is thus an urgent need for new vaccine manufacturing platforms that are able to rapidly and cheaply produce vaccine antigens. Platform technology based on *E. coli*, which can synthesize viral capsomeres at gram-per-litre levels, was developed by Middelberg et al. The high yield of capsid proteins or structural proteins (the basic unit of VLPs) could significantly reduce the time and the cost of vaccine production.^24^ A microbe-based platform has the potential to quickly provide affordable, safe, and efficacious vaccines in developing countries.

## *E. coli*-derived VLP vaccines and vaccine candidates

*E. coli* is the preferred recombinant expression host due to its ease of use and the low cost associated with cultivation. Several *E. coli*-derived VLP vaccines or vaccine candidates have entered clinical trials in recent years (Table 1). Hecolin, a p239 VLP-based vaccine, containing 368-606 aa of open reading frame 2 of a genotype 1 strain of HEV,^25^ was the first commercialized *E. coli*-derived vaccine for the prevention of HEV infection.^19^ Meanwhile, two *E. coli*-derived VLP-based vaccines (NCT01735006 and NCT02710851) against human papillomavirus (HPV) also has been developed in clinical trials. In addition, VLPs have been utilized as vaccine platforms to increase the immunogenicity of antigens. These chimeric VLP vaccines were both targeted against infectious and non-infectious diseases. The high-cost of the vaccines was the main limitation preventing worldwide implementation.^26^ Developing more affordable vaccines could partly address the inaccessibility and other hurdles faced by some commercial vaccines.

**Table 1:** *E. coli*-derived VLP based vaccines or vaccine candidates

Vaccine name	Company/Institution	VLP platform	Vaccine antigen	Clinical Trial/Approved	Reference or clinical trial identifier (NCT) ^*^
Prophylactic vaccines					
HEV Hecolin	Xiamen Innovax Biotech Co., Ltd (Xiamen, China)	HEV	HEV capsid polypeptide	Licensed	18, 19
HPV HPV16/18	Xiamen University, Xiamen Innovax Biotech Co., Ltd	HPV	HPV16/18 L1 major capsid protein	Phase III	NCT01735006
HPV6/11	Beijing Wantai Biological Pharmacy Enterprise Co., Ltd (Beijing, China)		HPV6/11 L1 major capsid protein	Phase II	NCT02710851
ACAM-FLU-A^a^	Sanofi Pasteur	HBcAg	Influenza A M2e	Phase I	NCT00819013
gH1-Qbeta^a^	A*STAR and Cytos Biotechnology	Bacteriophage Qβ	globular head domain (gH1) of haemagglutinin (HA)	Phase I	61
MalariVax (ICC-1132)^a^	Apovia	HBcAg	*Plasmodium falciparum* circumsporozoite protein	Phase I	NCT00587249
Therapeutic vaccines					
HBV ABX203 (HeberNasvac)^b^	The Center for genetic Engineering and Biotechnology, Cuba	HBV	HBsAg/HBcAg	Licensed	65, 66
Allergic rhinitis and asthma CYT003-QβG10^a^	Cytos Biotechnology	Bacteriophage Qβ	G10 (CpG DNA)	Phase II	NCT00890734
Malignant melanoma CYT004-MelQβG10^a^	Cytos Biotechnology	Bacteriophage Qβ	Melan-4, G10 DNA (CpG)	Phase II	NCT00651703
Alzheimer’s disease CAD106^a^	Cytos Biotechnology	Bacteriophage Qβ	Aβ1-6 epitope	Phase II	NCT01097096
Hypertension CYT006-AngQβ^a^	Cytos Biotechnology	Bacteriophage Qβ	Angiotensin II	Phase II	NCT00500786
Nicotine addiction NIC002^a^	Cytos Biotechnology	Bacteriophage Qβ	Nicotine hapten	Phase II	NCT01280968
Type II diabetes mellitus CYT013-IL1bQβ^a^	Cytos Biotechnology	Bacteriophage Qβ	IL-1β	Phase I	NCT00924105

^*^References or NCT numbers (registered at https://clinicaltrials.gov) are provided

^a^ Chimeric VLP-based vaccines: VLPs as vaccine platforms display heterologous epitopes or antigens on their surface by the way of genetic fusion or chemical conjugation

^b^ Hepatitis B virus surface antigen (HBsAg) and hepatitis B core antigen (HBcAg), were expressed in yeast (*Pichia pastoris*) and *E. coli,* respectively

### HEV vaccine

The HEV, the causative agent of hepatitis E, is the sole member of the genus *Hepevirus* within the family *Hepeviridae* and transmits primarily in a fecal-oral manner.^27, 28^ HEV infection is a serious threat to public health, especially in developing countries. Mammalian HEV is classified into four major genotypes, but only one serotype.^29^ This opens up the opportunity for the development of a univalent, broad-spectrum HEV vaccine. HEV is a 34-nm, non-enveloped, positive-sense single-stranded RNA icosahedral virus with an approximately 7.2-kb genome containing three open reading frames (ORFs). These ORFs encode a number of different proteins for various biological functions, among which ORF2 (660 amino acid) encodes the sole capsid protein, pORF2.^30^

**Fig. 2:**
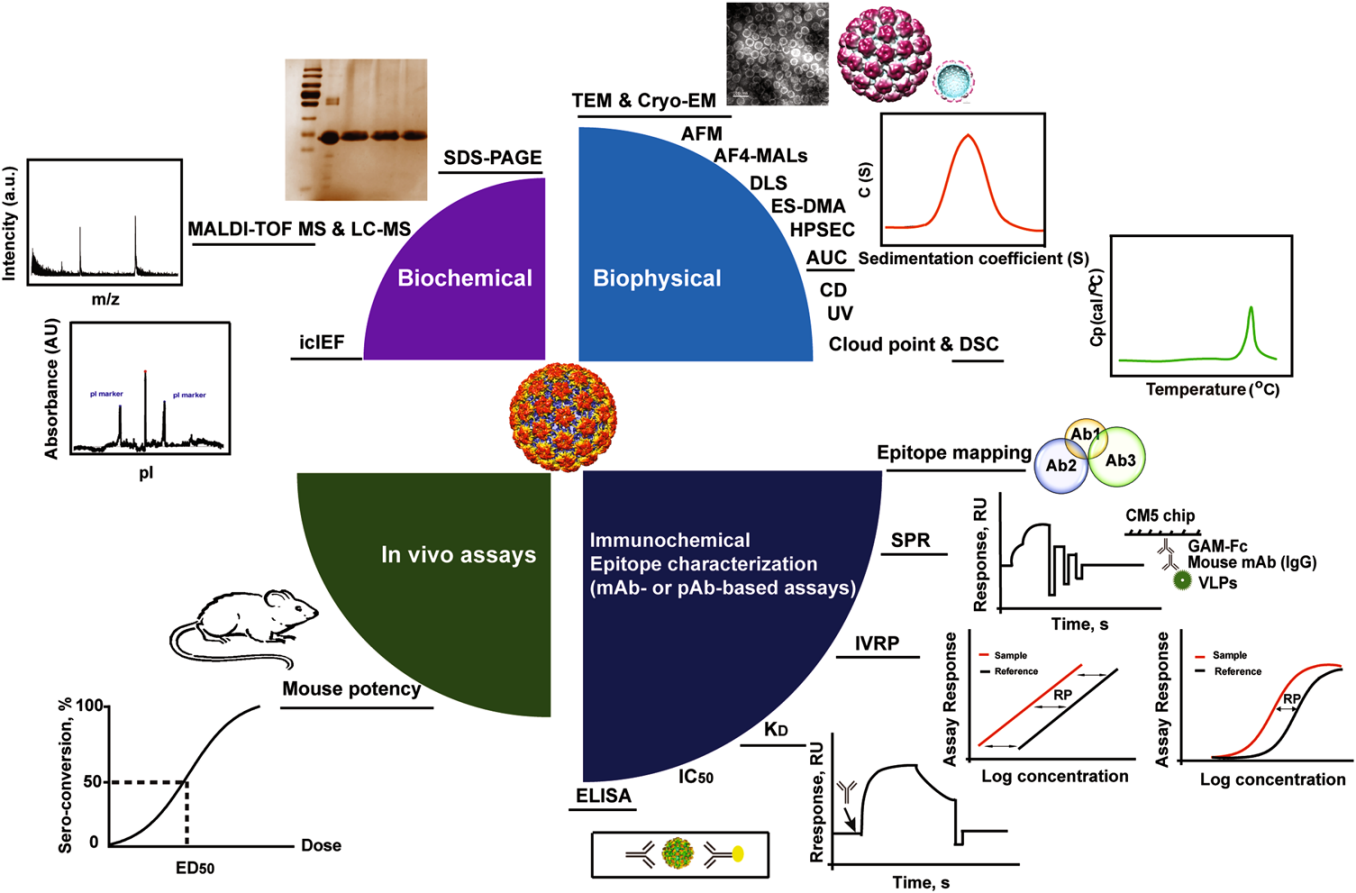
Analytical toolbox for the characterization of VLPs. A series of modern techniques make up a “toolbox” that has been extensively used for structural and functional characterization of VLPs. Biochemical: *SDS-PAGE* sodium dodecyl sulphate polyacrylamide gel electrophoresis, *MALDI-TOF MS* matrix-assisted laser desorption/ionization time of flight mass spectrometry,^33^ *LC-MS* liquid chromatography–mass spectrometry,^33^ *icIEF* imaged capillary isoelectric focusing has been widely used for protein characterization,^33^ Biophysical: the morphology of VLPs can be observed by TEM, Cry-EM, and AFM. *TEM* transmission electron microscopy, *Cry-EM* cry electron microscopy,^85^ *AFM* atomic force microscopy,^114^ AF4-MALS, DLS, ES-DMA, and HPSEC generally are used for the measurement the size of particles. *AF4-MALS* asymmetric flow field-flow fractionation coupled with multiple-angle light scattering,^117, 118^ *DLS* dynamic light scattering,^119^ *ES-DMA* electrospray differential mobility analysis,^118^ *HPSEC* high performance size exclusion chromatography,^33^ *AUC* analytical ultracentrifugation, *CD* Circular dichroism,^33^ *UV* ultraviolet spectroscopy,^33^ *DSC* differential scanning calorimetry, mAb or pAb-based assays are used to measure the concentration of functional epitopes in the vaccine samples. Epitope-mapping: comparable of epitope overlap of VLPs in different vaccine samples by mAbs; *SPR* surface plasmon resonance, *IVRP* in vitro relative potency, *KD* equilibrium dissociation constant,^114^ *IC*_*50*_ half maximal inhibitory concentration, *ELISA* enzyme-linked immunosorbent assay, The mini-VLP in the figure is the structure mode of HPV59, which was adapted from Structure, Li et al.^120^

Hecolin, the first prophylactic hepatitis E vaccine, was licensed in 2011 and launched in 2012 in China.^19^ It is also the world’s first *E. coli-*derived VLP-based vaccine synthesized on a commercial scale.^10^ The neutralizing and immunodominant epitopes from HEV genotype 1 were present on the surface of p239 VLPs.^9^ The high efficacy of the HEV vaccine was demonstrated by a randomized, double-blind, placebo-controlled phase III clinical trial;^31^ a follow-up study subsequently demonstrated long-term efficacy of up to 4.5 years after the initial vaccination.^32^ Product consistency was demonstrated through comprehensive characterization of antigens in Hecolin. The comparable p239 VLPs characteristics of the antigen produced at different scales indicated that the antigen manufacturing process was robust and scalable.^33^ The vaccine contains 30-μg truncated capsid protein formulated with aluminum adjuvants.^10^ Zhang et al. have demonstrated the preservation of critical antigen epitopes absorbed on adjuvants and recovered antigens post-dissolution treatment using a set of biochemical, biophysical, and immunochemical methods (Fig. 2).^34^ In that study, the anti-HEV monoclonal antibody 8C11, which was applied in different immunochemical methods, was able to capture the native HEV virions.^9^ This result indicated that virion-like epitopes are present on the surface of *E. coli*-derived p239 VLPs. Multi-year stability is required for marketed vaccines. The real-time and long-term stability of Hecolin, stored at 4 °C for 24 months, was evaluated using a set of biophysical, biochemical, and immunochemical approaches. The results demonstrated overall high structural stability of p239 VLPs over 24 months.^35^

### HPV vaccines

HPVs, non-enveloped double-stranded DNA viruses, are the causative agents of cervical cancer.^36, 37^ Papillomavirus virions consist of two structural proteins, L1 and L2; the major structural protein is L1, which is able to self-assemble into pentamers and subsequently into VLPs.^38^ Currently, three prophylactic HPV vaccines are based on VLPs, Gardasil-4 (a quadrivalent HPV16/18/6/11 vaccine produced in yeast), Gardasil-9 (a 9-valent 16/18/31/33/45/52/58/6/11 HPV vaccine produced in yeast), and Cervarix (a bivalent HPV 16/18 vaccine expressed via insect cells).^39–41^ Clinical trials have shown that all three vaccines consistently induced production of protective and neutralizing antibodies to prevent infection. These vaccines are generally well tolerated.^42, 43^ However, their high production and delivery costs are significant barriers to worldwide implementation.^44^ The globally licensed HPV vaccines, all produced in eukaryotic systems with high production cost,^45^ are thus excluded low-income regions, where cervical cancer results in higher mortality.^46^ Thus, there is a pressing need for more cost-effective vaccines.

Xiamen Innovax Biotech has used *E. coli* to produce a low-cost HPV vaccine based on L1 VLPs. L1 is the HPV major structural protein (the other minor capsid protein is L2).^47^ A bivalent HPV vaccine (Types 16, 18), based on these VLPs, has been developed and has been shown to be safe and highly immunogenic in preclinical studies. The data indicated that HPV16/18 VLPs were obtained from a prokaryotic expression system with desired immunogenicity.^48^ The results of a phase I safety trial showed that the *E. coli* expressed recombinant HPV 16/18 bivalent vaccine candidate is well tolerated in healthy women, with just few minor adverse events attributable to the vaccination were observed.^48^ The immunogenicity of vaccine was demonstrated in healthy young women in a phase II clinical trial.^49^ A large-scale phase III efficacy trial was initiated in November 2012 in China (NCT01735006). Additionally, another bivalent HPV vaccine candidate (Types 6, 11) obtained from *E. coli* is currently undergoing a phase II clinical trial (NCT02710851) (Table 1). The success of HPV L1 VLPs and HEV p239 obtained from *E. coli* indicates that this microbe-based vaccine technology may facilitate the development of cost-effective vaccines and bring benefits to people in developing countries.

### Other prophylactic vaccines

VLPs have been used as a vaccine delivery platform to increase the immunogenicity of antigens.^50^ Several chimeric VLP vaccine candidates are listed in Table 1. In addition to the HEV vaccine and HPV vaccines mentioned above, a malaria vaccine and two influenza VLP-based vaccines expressed by the *E. coli* system were reported.

#### Malaria vaccine

Malaria, caused by the *Plasmodium* parasite, is a serious public health problem in the tropics.^51^ There is no highly effective vaccine for malaria.^52^ A chimeric VLP-based vaccine candidate, MalariVax (ICC-1132), consists of a hepatitis B virus core VLPs produced in *E. coli*, displaying malaria epitopes (the *Plasmodium falciparum* circumsporozoite) on their surface. The results of a phase I trial showed clinical efficiency against malaria parasites.^53^ No subsequent clinical data were published.

#### Influenza vaccines

Due to viral drifts and shifts, a particular influenza vaccine cannot provide long-term immunity.^54^ A microbial platform may rapidly provide a vaccine to combat seasonal influenza epidemics.^55^ The anti-influenza A M2e-HBc vaccine candidate, ACAM-FLU-A, was produced by *E. coli*. Recombinant hepatitis B core antigen (HBcAg), as a carrier VLP, is one of the main structural antigens of HBV.^56^ The M2 external domain is a relatively conserved epitope in both human and avian influenza A viruses that is present on the surface of HBcAg VLPs. The immunogenicity has been confirmed in a phase I clinical trial (NCT00819013).^57–60^ In addition, globular head domain (gH1)-Qbeta, a fully bacterially produced influenza vaccine, was obtained by chemically conjugating the gH1 of hemagglutinin (HA) from the pandemic A/California/07/2009(H1N1) influenza strain to the Qbeta VLPs. A phase I trial has demonstrated that gH1-Qbeta was able to induce high titer of anti-viral antibodies with a favorable safety profile.^61^

### Therapeutic vaccines in clinical trials for human diseases

A combination vaccine used for chronic hepatitis B treatment, ABX203 (trade name HeberNasvac), is composed of hepatitis B virus surface (HBsAg) and core antigens (HBcAg), which are expressed in *Pichia pastoris* and *E. coli*, respectively.^62^ ABX203 has been shown to be effective and well tolerated in clinical trials.^63–65^ The Cuban regulatory authorities granted the Center for genetic Engineering and Biotechnology their first marketing authorization application for ABX203 in 2015.^66^ Additionally, a number of chimeric VLP vaccine candidates, chemically conjugated antigens to the RNA bacteriophage Qβ VLPs derived from *E. coli*, have been developed by Cytos Biotechnology AG (Switzerland) (Table 1). These chimeric VLP vaccine candidates are designed to targeted non-infectious diseases such as nicotine addiction, hypertension, cancer, diabetes, allergies, and Alzheimer’s disease. Results showed that the use of nicotine-Qβ VLPs, such as NIC002 (formerly CYT002-Nic002), have promoted long-term abstinence from smoking.^67, 68^ Similarly, a Qβ VLP conjugated with a modified Ang II peptide, CYT006-AngQβ, were developed as an anti-hypertensive vaccine.^69^ Additionally, CYT004-MelQβG10 (NCT00651703), CYT103-IL1bQβ (NCT00924105), and CYT003-QβG10 (NCT00890734), which are directed against malignant melanoma, Type II diabetes, allergic rhinitis, and asthma, respectively, are currently in various stages of clinical trials. CAD-106, in which Qβ VLP is covalently coupled to the Aβ1-6 peptide, is an immunotherapeutic vaccine for Alzheimer’s disease currently undergoing a Phase II trial.^70–72^

In addition, many *E. coli*-derived VLP-based vaccine candidates, against West Nile virus,^73^ foot-and-mouth disease virus^74^ and hepatitis C virus^75^ also have been developed in preclinical studies. The potency of these *E. coli*-derived VLP antigens has been demonstrated in different animal models. The efficacy and safety of a vaccine need to be demonstrated for licensing in human use.^76, 77^ Post licensure, the quality of vaccines during manufacturing and storage should be assessed to ensure their safety and efficacy throughout the life cycle management of vaccine commercialization. Structural and functional assessment of VLPs is the most critical antigen characterization assays for recombinant protein based vaccines.

## Structural and functional assessment of VLPs

*E. coli*-derived HEV p239 VLPs and HPV VLPs consist only of the viral capsid protein without incorporating genetic materials but retain a conformation similar to that of the native virus.^9, 78^ Generation of functional antibodies is dependent on the correct antigen conformation and native-like epitopes being present on the surface of VLPs.^79^ VLPs containing virion-like epitopes can be acquired via antigen-presenting cells and then induce a protective humoral immune response.^50, 80^ Thus, recombinant VLPs must be correctly folded to ensure their function by inducing a protective humoral immune response. The spatial or higher-order structure of the vaccine antigen is the basis of the various biological functions of protein-based vaccines. Quantitative analysis of the functional epitopes on VLPs using monoclonal antibody-based assays can be an advantageous way to ensure vaccine safety and efficacy.^81^ Multifaceted analytical approaches, such as biochemical, biophysical, and immunochemical methods (Fig. 2), have been well established and are widely used for the evaluation of three different licensed recombinant VLP-based vaccines: hepatitis B vaccine, hepatitis E vaccine, and HPV vaccine.^82^

The identification of the primary structure indicated that the target protein composed of VLPs was successfully expressed. Biochemical characterization includes the primary amino acid sequence, molecular weight, isoelectric point, and purity of the VLPs.^10^ The secondary and tertiary structures of the VLPs can be measured by circular dichroism and ultraviolet spectroscopy.^33^ Mass spectrometry is an indispensable analytical technique used to determine the mass of proteins and their amino acid composition.^33, 83, 84^ This tool is useful for process monitoring and demonstrating final product consistency at single amino acid level. SDS-PAGE, the most common method that used to determine the purity, integrity and molecular weight of the purified antigen.^25, 84^ The morphological characteristics and the state of the VLP are amenable to imaging by transmission electron microscopy (TEM),^85^ analytical ultracentrifugation (AUC) or density gradient ultracentrifugation.^86, 87^ TEM methods can be used to determine the three-dimensional structure of VLPs and investigate their interaction with antibodies or their appearance when adsorbed to an adjuvant, in combination with modern computational tools, bioinformatics, homology modeling, docking, and MD simulation.^20, 85, 88^ Differential scanning calorimetry and cloud point have been widely applied for investigating the thermal stability and aggregation propensity of recombinant proteins.^33, 89–91^ These modern techniques make up a “toolbox” that has been extensively used for structural and functional characterization of VLP-based vaccines (Fig. 2).

As an immunogen, VLP-based vaccines generally stimulate a humoral and a mostly CD4 T cell-mediated immune response.^80^ VLPs were injected into individuals to develop protective immunity against infection. Neutralizing and immunodominant epitopes on antigen are the structural basis of an epitope to elicit functional and protective antibodies.^79^ The functional epitopes can be quantitated by their ability to bind to a panel of specific monoclonal antibodies.^92–94^ Monoclonal antibodies have been developed as specific probes to identify and characterize virion-like epitopes.^34, 95, 96^ Binding activity to a certain neutralizing epitope can serve as an excellent surrogate marker for in vivo immunogenicity or vaccine efficacy. Currently, various immunoassays have been applied for assessment of the antigenicity of HBV, HPV, and HEV VLPs using a panel of specific and functional monoclonal antibodies (Fig. 2). These methods include one-site binding and label-free SPR technology,^33, 88, 97^ solution competition ELISA (IC_50_),^88, 98, 99^ and sandwich ELISA.^95, 100, 101^ In vitro relative potency assays (IVRP assays) generally is a sandwich-type immunoassay that uses neutralizing monoclonal antibodies to measure the concentration of functional epitopes in the vaccine sample. The IVRP assay has been shown to have a good correlation with mouse potency in Gardasil-4.^101^ Thus, mouse potency can be replaced by IVRP for release and stability testing, as well as monitoring of the production process.

## Discussion

VLPs have been widely used in vaccinology. The next generation of VLP-based vaccine candidates must be creative in form and function to satisfy diverse needs.^50^ Several viral structures produced in *E. coli*, such as HBcAg, Qβ, AP250, murine polyomavirus and HPV, have been used for vaccine platforms.^22, 59, 69, 102, 103^ Vaccinologists can now add heterologous epitopes or antigens to these VLPs from different origins achieved by genetically fusing or chemical conjugation.^104^ Middelberg et al. have developed in vitro cell-free assembly of modular VLPs based on murine polyomavirus capsid proteins expressed in *E. coli* as vaccine carriers to enhance immune responses, especially to weakly or non-immunogenic antigens.^22^ These modular VLPs have potential for use as a vaccine platform to increase the efficacy and stability and to allow for more versatile display of antigens. Re-engineering or grafting epitopes in chimeric VLPs may widen the coverage spectrum compared to monovalent vaccines.^50^ Several chimeric VLP vaccine candidates have been developed by chemically conjugating foreign antigens to RNA bacteriophage Qβ VLPs obtained from *E. coli*. These chimeric VLP vaccines are currently in clinical development (Table 1). The yield of Qβ VLP production in *E. coli* is higher than that in yeast.^105^ RNA bacteriophage VLPs naturally encapsidated ssRNA in *E. coli*, such that it could influence the immune bias when used in mouse immunizations, with a shift from IgG1 to IgG2a compared to VLPs without RNA, indicating that the Th1-biased immune response has occurred.^106–108^ The Th1-type immune response is essential for the control of intracellular pathogens and could be an ideal platform for future prophylactic (malaria, HIV, Herpes viruses) and therapeutic vaccine applications (cancer and chronic hepatitis).^109^ VLPs derived from a given viral protein or as displaying bionanoparticles of foreign epitopes could enhance the immunogenicity of the B-cell epitopes on the particle surface or modulate the Th1- and/or Th2-immune response due to the the nature of the B-cell or T-cell epitopes built in via recombinant technology as part of the protein-based particles. 

The commercial VLP-based vaccines have been constructed through eukaryotic or prokaryotic systems. A brief comparison among different expression systems with respect to their applications in producing recombinant VLPs has been summarized in Table 2. As a manufacturing platform, *E. coli* faced several obstacles, which may limit its application in protein-based biopharmaceuticals. Their limitation factors included: (1) lack of ability to produce the correct disulfide bonds, (2) fail to produce recombinant proteins with mammalian-like post-translational modifications, (3) the problems of protein solubility, and (4) the presence of endotoxins (lipopolysaccharide, LPS).^17^ Post-translational modifications play an important role in protein folding, processing, and stability, as well as final biological activity and even the immunogenicity of the protein.^110^ Disulfide bond formation, glycosylation, phosphorylation and proteolysis processing play a crucial role in biological activity of some recombinant proteins.^15^ *E. coli* cannot synthesize useful HBsAg particles, probably because of unfavorable environmental conditions, such as pH and redox potential, or lipid compositions within the bacterium.^111^ Studies have also shown that HBcAg phosphorylation is essential for viral replication and capsid formation.^112^ Therefore, the expressed proteins may be insoluble, unstable or inactive without post-translational modifications. *E. coli* stands out as the expression system for the production of small recombinant proteins without post-translational modifications.^14, 15^ However, Spiess et al. developed an approach for the efficient generation of nonimmunogenic, stable bispecific antibodies with a natural IgG architecture by co-culture of bacteria (*E. coli*) expressing two distinct half-antibodies.^113^ This technology provides a rapid generation of biospecific antibodies with natural architecture from any two existing antibodies for academic research and industrial development.

**Table 2:** A brief comparison among different systems with respect to their applications in producing recombinant VLPs

Property	*E. coli*	Yeast	Baculovirus-insect cells	Mammalian cells
Production cost	+	++	+++	++++
VLP production levels	++++	+++	++	+
VLP complexity^20^	+	++	++++	++
Post-translational modifications(PTMs)*				
Disulfide bond	Unfavorable redox potential for disulfide bond formation	Yes	Yes	Yes
O-glycosylation	No	Yes	Yes	Yes
N-glycosylation	No	Yes	The inability to synthesize mammalian-type N-glycans	Yes
Phosphorylation	No	Yes	Yes	Yes
Acylation	No	Yes	Yes	Yes
γ-Carboxylation	No	No	No	Yes
Applications**	Simple polypeptides and proteins (Hecolin)	Mammalian-like or secreted proteins (Gardasil-4 and Gardasil-9)	Mammalian-like or secreted proteins (Cervarix)	Mammalian proteins (GenHevac B)

*Post-translational modifications (PTMs) are similar or identical to those occurring in mammalian cells

**The application examples of VLP-based vaccines derived from different expression systems were summarized in Fig. 1 and Supplementary Table 1S. Hecolin (HEV vaccine): manufactured by Xiamen Innovax Biotech Co., Ltd. Gardasil-4 and Gardasil-9 (HPV vaccines): manufactured by Merck. Cervarix (HPV vaccine): manufactured by GSK. GenHevac B (HBV vaccine): manufactured by Pasteur-Merieux Aventis

Currently, numerous mutant *E. coli* strains have been developed to improve the different protein expression (representative examples are shown in Table 3). Origami™ 2(DE3) is mutated in glutathione reductase and thioredoxin reductase to promote target protein disulfide bond formation. Several strategies were applied to solve the insolubility of proteins by adjusting culture conditions (such as low temperature), using fusion protein systems and in vitro denaturing/re-assembly of insoluble inclusion body.^17^ The key for VLPs to elicit both humoral and CD4 T cell-mediated immune responses is that the VLPs retain a conformation similar to that of native viruses in molecular scaffolds. Zhao et al. have demonstrated that disassembly-reassembly (D/R) of HPV VLPs produced more virion-like antibody reactivity.^114^ The D/R treatment with defined and controlled physicochemical conditions have been explored for better folding of structural proteins, such as the formation of correct disulfide bond.^87^ These additional bioprocessing steps with well-controlled conditions will be essential to obtain more desirable VLPs with in-particle assembly and particle-to-particle homogeneity. Most gram-negative bacteria contain endotoxins, LPSs, which can induce a pyrogenic response.^115^ Thus, for safe use in humans, LPSs must be removed from the recombinant proteins expressed in *E. coli* to maintain the levels of endotoxins below a certain threshold. However, the removal of LPSs increases the complexity and the cost of protein purification processes.^115^ Recently, Mamat et al. constructed endotoxin-free *E. coli* strains by multi-step mutagenesis, KPM335, which provided an endotoxin-free environment and can be a versatile expression system for protein production.^115, 116^ Thus, although *E. coli* has some limitations, it could be a potential expression host for rapid, scalable and economical VLP-based vaccine production.

**Table 3:** Representative recombinant *E. coli* strains for protein expression

Strains	Description	Applications	Company/Institution
**BL21**	Deficient in both *Lon* and *OmpT* proteases	General purpose expression host	Novagen
**C2528 (Lemo21(DE3) Competent** ***E. coli*** **)**	Deficient in *Lon* and *OmpT* proteases, T7 expression strain, resistant to phage T1 (*fhuA2*), tunable expression by lysozyme (lysY)	Expression membrane proteins, toxic proteins and proteins prone to insoluble expression	New England Biolabs
**BL21 Star™(DE3) pLysS**	RNaseE (*rne*131) mutant	Ideal for high-level expression of non-toxic but potentially growth-inhibiting recombinant proteins	Invitrogen
**Origami™ 2(DE3)**	Mutations in glutathione reductase (*gor*) and thioredoxin reductase (*trxB*)	Disulfide-bonded protein expression	Novagen
**C2529 (NiCo21(DE3) Competent** ***E. coli*** **)**	Mutation at GlmS, target proteins carrying an intein-chitin binding domain (intein-CBD) tag/ Poly-histidine tag	Improved purity of target proteins isolated by immobilized chitin affinity chromatography/ immobilized metal affinity chromatography	New England Biolabs
**KPM335**	Mutations in *ΔkdsD*, *ΔgutQ*, *ΔlpxL*, *ΔlpxM*, *ΔpagP*, *ΔlpxP*, *ΔeptA*, *msbA52* and *frr181*	Deficient in synthesis of LPS, endotoxin free strains for proteins expression	Research Corporation Technologies

## Conclusions

In summary, several promising *E. coli*-derived VLP-based vaccines or vaccine candidates, directed against both infectious and non-infectious diseases, have been currently commercialized or are being developed in the clinical testing stage. The success of *E. coli*-derived VLPs (Hecolin) as recombinant vaccine antigens suggested that using microbial synthesis has the potentials to facilitate the production of low-cost vaccines for global use. VLPs mimicking viral capsids or chimeric VLPs by grafting epitopes of interests to a well-behaved VLP display vector are platforms for future vaccines via structure-based modular design. Different analytical methods for antigen characterization provide important supports for recombinant VLP-based vaccines to ensure their efficacy and safety, and most importantly the preservation of native-like epitopes during manufacturing, storage and transportation of the vaccines. Further improvements on the *E. coli* platform could be achieved by genetically modify the expression host for achieving certain specific goals, such as protein expression with post-translational modifications. Better understanding of protein production and self-assembly would facilitate scale up and better process control at commercial production scale. As a result, rapid and inexpensive VLP-based vaccine production could be realized for global accessibility.

## Electronic supplementary material

Supplementary Information
