# Supplementary material for: Escherichia coli-derived virus-like particles in vaccine development
Source: NPJ Vaccines. 2017 Feb 9;2:3. doi: 10.1038/s41541-017-0006-8 (PMC5627247; doi:10.1038/s41541-017-0006-8)
Supplement: Supplementary file 1 — Supplementary Information [file 41541_2017_6_MOESM1_ESM.docx]

**Supplementary Information**

**Table 1S.** VLP-based human vaccines or vaccine candidates in clinical development

| **Vaccine name** | **Company/Institution** | **Expression system** | **Vaccine antigen** | **Status** | **Reference or clinical**  **trial identifier (NCT)*** |
| --- | --- | --- | --- | --- | --- |
| ***Hepatitis B*** | | | | | |
| Recombivax HB^®^ | Merck | Yeast (*S. cerevisiae*) | HBsAg | Licensed | [1](#_ENREF_1),[2](#_ENREF_2),[3](#_ENREF_3),[4](#_ENREF_4) |
| Engerix-B^®^ | GSK | Yeast (*S. cerevisiae*) | HBsAg | Licensed | [1](#_ENREF_1),[5](#_ENREF_5),[6](#_ENREF_6) |
| Fendrix^®^ | GSK | Yeast (*S. cerevisiae*) | HBsAg | Licensed | [7](#_ENREF_7),[8](#_ENREF_8) |
| DTP-Hep B^®^ | P.T. Bio Farma | Yeast (*P. pastoris*) | HBsAg | Licensed | [9](#_ENREF_9) |
| Enivac HB^®^ | Panacea Biotec | Yeast (*P. pastoris*) | HBsAg | Licensed | [10](#_ENREF_10),[11](#_ENREF_11) |
| Euvax B^®^ | LG Life Sciences | Yeast (*S. cerevisiae*) | HBsAg | Licensed | [12](#_ENREF_12),[13](#_ENREF_13) |
| GeneVac-B^®^ | Serum Inst. of India | Yeast (*H. polymorpha*) | HBsAg | Licensed | [14](#_ENREF_14),[15](#_ENREF_15) |
| Heberbiovac HB^®^ | CIGB-Heber Biotec | Yeast (*P. pastoris*) | HBsAg | Licensed | [16](#_ENREF_16),[17](#_ENREF_17) |
| Hepavax-Gene^®^ | GreenCross Vaccine Corporation | Yeast (*H. polymorpha*) | HBsAg | Licensed | [18](#_ENREF_18),[19](#_ENREF_19) |
| Revac-B^®^ | Bharat Biotech International | Yeast (*P. pastoris*) | HBsAg | Licensed | [20](#_ENREF_20) |
| Shanvac-B^®^ | Shantha Biotechnics | Yeast (*P. pastoris*) | HBsAg | Licensed | [21](#_ENREF_21) |
| Butang | Butantan Institute | Yeast (*H. polymorpha*) | HBsAg | Licensed | [22](#_ENREF_22),[23](#_ENREF_23) |
| Quinvaxem^®^ | Crucell | Yeast (*H. polymorpha*) | HBsAg | Licensed | [24](#_ENREF_24),[25](#_ENREF_25) |
| Bio-Hep-B^®^ (Sci-B-Vac^®^) | BTG (SciGen, FDS Pharma) | Mammalian (Chinese hamster ovary cells) | HBsAg | Licensed | [26](#_ENREF_26) |
| GenHevac B^®^ | Pasteur-Merieux Aventis | Mammalian (Chinese hamster ovary cells) | HBsAg | Licensed | [27](#_ENREF_27),[28](#_ENREF_28),[29](#_ENREF_29) |
| ABX203 (HeberNasvac) | CIGB | Yeast (*P. pastoris*) & Bacteria (*E. coli*) | HBsAg/HBcAg | Licensed | [30](#_ENREF_30),[31](#_ENREF_31) |
|  | Polish Academy of Sciences/ Thomas Jefferson University | Plant (Transgenic lettuce) | HBsAg | Phase 1 | [32](#_ENREF_32) |
|  | ASA | Plant (Transgenic potato) | HBsAg | Phase 1 | [33](#_ENREF_33) |
| ***Influenza*** | | | | | |
|  | Novavax | Insect (Sf-9 cells) | Influenza(A/California/04/09 (H1N1) HA, NA) | Phase 2 | NCT01072799 |
| **Vaccine name** | **Company/Institution** | **Expression system** | **Vaccine antigen** | **Status** | **Reference or clinical**  **trial identifier (NCT)*** |
|  | Novavax | Insect (Sf-9 cells) | Influenza(A/Brisbane/59/07 (H1N1), A/Brisbane/10/07, B/Florida/04/06 (H3N2) HA, NA) | Phase 2 | NCT00903552 |
|  | Novavax | Insect (Sf-9 cells) | Influenza(A/Indonesia/05/05  (H5N1) HA, NA) | Phase 2 | NCT00519389 |
|  | Novavax | Insect (Sf-9 cells) | A/Anhui/1/13-like A(H7N9) |  | NCT01897701 |
|  | Novavax | Insect (Sf-9 cells) | Trivalent Seasonal Influenza Virus-Like Particle (VLP) Vaccine | Phase 2 | NCT00903552 |
|  | Novavax | Insect (Sf-9 cells) | Quadrivalent Seasonal Influenza Virus-Like Particle(VLP) Vaccine |  | NCT01561768 |
|  | Medicago | Plant (transient N. benthamiana) | Influenza(A/Indonesia/05/05 (H5N1) HA) | Phase 2 | NCT01244867 |
|  | Medicago | Plant (transient N. benthamiana) | Influenza(A/California/04/09 (H1N1) HA) | Phase 1 | NCT01302990 |
|  | Medicago | Plant (transient N. benthamiana) | Influenza H7N9 A/Hangzhou/1/13 | Phase 1 | NCT02022163 |
|  | Medicago | Plant (transient N. benthamiana) | Influenza (A/California/07/2009 H1N1 (A/H1N1 Cal), A/Victoria/361/11 H3N2 (A/H3N2 Vic), B/Brisbane/60/08 (B/Bris, Victoria lineage) or B/Wisconsin/1/10 (B/Wis, amagata lineage) | Phase 2 | NCT02233816 |
|  | Cytos and A*STAR | Bacteria (*E. coli*) | A/California/07/2009(H1N1) (gH1-Qbeta) | Phase 1 | [34](#_ENREF_34) |
| ACAM-FLU-A | Sanofi Pasteur | Bacteria (*E. coli*) | Influenza A M2e | Phase 1 | NCT00819013 |
| **Human papillomavirus** | | | | | |
| Gardasil-4 | Merck | Yeast (*S. cerevisiae*) | HPV6/11/16/18 L1 | Licensed | [35](#_ENREF_35) |
| Cervarix^®^ | GSK | Insect (High Five^TM^ cells) | HPV16/18 L1 | Licensed | [36](#_ENREF_36) |
| Gardasil-9 | Merck | Yeast (*S. cerevisiae*) | HPV6/11/16/18/31/33/45/52/58 L1 | Licensed | [37](#_ENREF_37) |
| **Vaccine name** | **Company/Institution** | **Expression system** | **Vaccine antigen** | **Status** | **Reference or clinical**  **trial identifier (NCT)*** |
| HPV16/18 | Xiamen Innovax Biotech Co., Ltd | Bacteria (*E. coli*) | HPV16/18 L1 | Phase 3 | NCT01735006 |
| HPV16/18 | Shanghai Zerun Biotechnology Co., Ltd | Yeast (*S. cerevisiae*) | HPV16/18 L1 | Phase 1 | NCT02733068 |
| HPV6/11 | Xiamen Innovax Biotech Co., Ltd | Bacteria (*E. coli*) | HPV6/11 L1 | Phase 2 | NCT02710851 |
| **Norwalk virus** | | | | | |
|  | Baylor College of Medicine | Insect (Sf-9 cells) | Norwalk virus coat protein | Phase 1 | [38](#_ENREF_38) |
|  | LigoCyte Pharmaceuticals | Insect (Sf-9 cells) | Norwalk virus coat protein | Phase 1 | NCT00806962 |
|  | Center for Vaccine Development (University of Maryland) | Insect (Sf-9 cells) | Norwalk virus coat protein | Phase 1 | [39](#_ENREF_39) |
| Norovirus Bivalent-Vaccine | Takeda | Insect (Sf-9 cells) | Norwalk virus coat protein | Phase 2 | NCT01609257 |
| **Malaria (*P. falciparum*)** | | | | | |
| MalariVax  (ICC-1132) | Apovia | Bacteria (*E. coli*) | *Plasmodium falciparum* circumsporozoite protein | Phase 1 | NCT00587249 |
| RTS,S | GSK/PATH Malaria Vaccine Initiative & Gates Found | Yeast (*S. cerevisiae*) | *Plasmodium falciparum* circumsporozoite protein | Phase 3 | NCT00872963 |
| **Hepatitis E** | | | | | |
| Recombinant (Sar 56 kDa) | GSK | Insect cells | HEV capsid polypeptide (56-kDa protein) | Phase 2 | NCT00287469 |
| Hecolin^®^ | Xiamen Innovax Biotech Co., Ltd | Bacteria (*E. coli*) | HEV capsid polypeptide( HEV 239) | Licensed | [40](#_ENREF_40),[41](#_ENREF_41),[42](#_ENREF_42),[43](#_ENREF_43) |
| **Human immunodeficiency virus** | | | | | |
| HIV | British Biotech Pharmaceuticals/ NIAID | Yeast (*S. cerevisiae*) | HIV-1 Gag p17/p24 | Phase 2 | [44](#_ENREF_44) |
| **Vaccine name** | **Company/Institution** | **Expression system** | **Vaccine antigen** | **Status** | **Reference or clinical**  **trial identifier (NCT)*** |
| **Allergic rhinitis and asthma** | | | | | |
| CYT003-QβG10 | Cytos Biotechnology | Bacteria (*E. coli*) | G10 (CpG DNA) | Phase 2 | NCT00890734 |
| **Alzheimer’s disease** | | | | | |
| CAD106 | Cytos Biotechnology | Bacteria (*E. coli*) | Aβ1-6 | Phase 2 | NCT01097096 |
| **Hypertension** | | | | | |
| CYT006-AngQβ | Cytos Biotechnology | Bacteria (*E. coli*) | Angiotensin II | Phase 2 | NCT00500786 |
| **Malignant melanoma** | | | | | |
| CYT004-MelQβG10 | Cytos Biotechnology | Bacteria (*E. coli*) | Melan-4, G10 DNA (CpG) | Phase 2 | NCT00651703 |
| **Type II diabetes mellitus** | | | | | |
| CYT013-IL1bQβ | Cytos Biotechnology | Bacteria (*E. coli*) | IL-1β | Phase 1 | NCT00924105 |
| **Nicotine addiction** | | | | | |
| NIC002 | Cytos Biotechnology | Bacteria (*E. coli*) | Nicotine | Phase 2 | NCT01280968 |
| **Human parvovirus B19** | | | | | |
| VAI-VP705 | NIH/Meridian Life Science | Insect (Sf-9 cells) | B19 VP1, VP2 | Phase 2 | NCT00379938 |
| **Rabies** | | | | | |
|  | Thomas Jefferson University | Plant (Transgenic spinach) | Rabies GP/NP | Phase 1 | [45](#_ENREF_45) |
| **Respiratory syncytial virus** | | | | | |
|  | Novavax | Insect (Sf-9 cells) | RSV F protein | Phase 3 | NCT02624947 |

*****References or NCT numbers (registered at <https://clinicaltrials.gov>) are provided.

**Supplementary References**

1. Stephenne, J. Recombinant versus plasma-derived hepatitis B vaccines: issues of safety, immunogenicity and cost-effectiveness. *Vaccine* **6**, 299-303 (1988).

2. Venters, C., Graham, W. & Cassidy, W. Recombivax-HB: perspectives past, present and future. *Expert review of vaccines* **3**, 119-129 (2004).

3. Zhao, Q.*, et al.* In-depth process understanding of RECOMBIVAX HB(R) maturation and potential epitope improvements with redox treatment: multifaceted biochemical and immunochemical characterization. *Vaccine* **29**, 7936-7941 (2011).

4. Rustandi, R.R., Wang, F., Hamm, C., Cuciniello, J.J. & Marley, M.L. Development of imaged capillary isoelectric focusing method and use of capillary zone electrophoresis in hepatitis B vaccine RECOMBIVAX HB(R). *Electrophoresis* **35**, 1072-1078 (2014).

5. Andre, F.E. & Safary, A. Summary of clinical findings on Engerix-B, a genetically engineered yeast derived hepatitis B vaccine. *Postgraduate medical journal* **63 Suppl 2**, 169-177 (1987).

6. Keating, G.M. & Noble, S. Recombinant hepatitis B vaccine (Engerix-B): a review of its immunogenicity and protective efficacy against hepatitis B. *Drugs* **63**, 1021-1051 (2003).

7. de Silva, T.I.*, et al.* Successful use of Fendrix in HIV-infected non-responders to standard hepatitis B vaccines. *The Journal of infection* **68**, 397-399 (2014).

8. Fabrizi, F. & Martin, P. A new single-dose hepatitis B vaccine versus a conventional vaccine in patients with end-stage renal disease. *Nat Clin Pract Neph* **2**, 356-357 (2006).

9. Bar-On, E.S.*, et al.* Combined DTP-HBV-HIB vaccine versus separately administered DTP-HBV and HIB vaccines for primary prevention of diphtheria, tetanus, pertussis, hepatitis B and Haemophilus influenzae B (HIB). *The Cochrane database of systematic reviews*, Cd005530 (2009).

10. Hussain, Z.*, et al.* Evaluation of immunogenicity and reactogenicity of recombinant DNA hepatitis B vaccine produced in India. *World journal of gastroenterology* **11**, 7165-7168 (2005).

11. Kaur, H. & Mani, A. Seroprotection following Enivac-HB, a recombinant hepatitis B vaccine. *Indian journal of gastroenterology : official journal of the Indian Society of Gastroenterology* **19**, 41 (2000).

12. Brandao, A.*, et al.* Efficacy of a recombinant hepatitis B vaccine (Euvax-B) in adult patients awaiting liver transplantation: preliminary results. *Transplantation proceedings* **31**, 3055-3056 (1999).

13. Tele, S.A.*, et al.* Immunogenicity of a recombinant hepatitis B vaccine (Euvax-B) in haemodialysis patients and staff. *European journal of epidemiology* **17**, 145-149 (2001).

14. Shivananda, Somani, V., Srikanth, B.S., Mohan, M. & Kulkarni, P.S. Comparison of two hepatitis B vaccines (GeneVac-B and Engerix-B) in healthy infants in India. *Clinical and vaccine immunology : CVI* **13**, 661-664 (2006).

15. Kulkarni, P.S.*, et al.* Immunogenicity of a new, low-cost recombinant hepatitis B vaccine derived from Hansenula polymorpha in adults. *Vaccine* **24**, 3457-3460 (2006).

16. Galban Garcia, E.*, et al.* Field trial of the Cuban recombinant vaccine against hepatitis B (Heberbiovac HB). Study in newborn infants born to AgsHB+ mothers]. *Revista cubana de medicina tropical* **44**, 149-157 (1992).

17. Diaz Gonzalez, M., Navia Molina, O., Bravo Gonzalez, J.R., Pedroso Flaquet, P. & Urbino Lopez, A. The reactogenicity of Heberbiovac-HB vaccine at different doses. *Revista cubana de medicina tropical* **47**, 65-70 (1995).

18. Hieu, N.T., Kim, K.H., Janowicz, Z. & Timmermans, I. Comparative efficacy, safety and immunogenicity of Hepavax-Gene and Engerix-B, recombinant hepatitis B vaccines, in infants born to HBsAg and HBeAg positive mothers in Vietnam: an assessment at 2 years. *Vaccine* **20**, 1803-1808 (2002).

19. Rebedea, I., Diaconescu, I.G., Bach, D., Bartelsen, O. & Arndtz, N. Comparison of thiomersal-free and thiomersal-containing formulations of a recombinant hepatitis B vaccine (Hepavax-Gene) in healthy adults. *Vaccine* **24**, 5320-5326 (2006).

20. Lakshmi, G., Reddy, R.P., Kumar, K.K., Bhavani, N.V. & Dayanand, M. Study of the safety, immunogenicity and seroconversion of a hepatitis-B vaccine in malnourished children of India. *Vaccine* **18**, 2009-2014 (2000).

21. Abraham, P.*, et al.* Evaluation of a new recombinant DNA hepatitis B vaccine (Shanvac-B). *Vaccine* **17**, 1125-1129 (1999).

22. Ioshimoto, L.M.*, et al.* Safety and immunogenicity of hepatitis B vaccine ButaNG in adults. *Revista do Instituto de Medicina Tropical de Sao Paulo* **41**, 191-193 (1999).

23. Isolani, A.P., Sversuti, C.S., Sell, A.M. & Moliterno, R.A. Protection against hepatitis B by the Butang recombinant vaccine in newborn children in South Brazil. *Memorias do Instituto Oswaldo Cruz* **101**, 551-553 (2006).

24. Schmid, D.A., Macura-Biegun, A. & Rauscher, M. Development and introduction of a ready-to-use pediatric pentavalent vaccine to meet and sustain the needs of developing countries--Quinvaxem(R): the first 5 years. *Vaccine* **30**, 6241-6248 (2012).

25. Aspinall, S., Traynor, D., Bedford, P. & Hartmann, K. Lot-to-lot consistency study of the fully liquid pentavalent DTwP-HepB-Hib vaccine Quinvaxem ((R)) demonstrating clinical equivalence, suitability of the vaccine as a booster and concomitant administration with measles vaccine. *Human vaccines & immunotherapeutics* **8**, 1109-1118 (2012).

26. Hepatitis B vaccine recombinant (bio-technology general). BioHepB, Sci-B-Vac. *Drugs in R&D* **2**, 199-200 (1999).

27. Soulie, J.C.*, et al.* Immunogenicity and safety in newborns of a new recombinant hepatitis B vaccine containing the S and pre-S2 antigens. *Vaccine* **9**, 545-548 (1991).

28. Michel, M.L. & Tiollais, P. Hepatitis B vaccines: protective efficacy and therapeutic potential. *Pathologie-biologie* **58**, 288-295 (2010).

29. Assateerawatt, A., Tanphaichitr, V.S., Suvatte, V. & Yodthong, S. Immunogenicity and efficacy of a recombinant DNA hepatitis B vaccine, GenHevac B Pasteur in high risk neonates, school children and healthy adults. *Asian Pacific journal of allergy and immunology / launched by the Allergy and Immunology Society of Thailand* **11**, 85-91 (1993).

30. Lobaina, Y.*, et al.* Demonstration of safety, immunogenicity and evidences of efficacy of the therapeutic vaccine candidate HeberNasvac and characterization of chronic hepatitis B patient populations. *Biotecnología Aplicada*, 32:3511-3513 (2015).

31. ABX203 (HeberNasvac) Granted Cuban Marketing Authorization to Treat Chronic Hepatitis B. Available at: <http://www.abivax.com/images/pdf/151208_ABX203_Cuban_Authorization.pdf> (2015).

32. Kapusta, J.*, et al.* A plant-derived edible vaccine against hepatitis B virus. *FASEB journal : official publication of the Federation of American Societies for Experimental Biology* **13**, 1796-1799 (1999).

33. Thanavala, Y.*, et al.* Immunogenicity in humans of an edible vaccine for hepatitis B. *Proceedings of the National Academy of Sciences of the United States of America* **102**, 3378-3382 (2005).

34. Low, J.G.*, et al.* Safety and immunogenicity of a virus-like particle pandemic influenza A (H1N1) 2009 vaccine: results from a double-blinded, randomized Phase I clinical trial in healthy Asian volunteers. *Vaccine* **32**, 5041-5048 (2014).

35. McLemore, M.R. Gardasil: Introducing the new human papillomavirus vaccine. *Clinical journal of oncology nursing* **10**, 559-560 (2006).

36. Monie, A., Hung, C.F., Roden, R. & Wu, T.C. Cervarix: a vaccine for the prevention of HPV 16, 18-associated cervical cancer. *Biologics : targets & therapy* **2**, 97-105 (2008).

37. Printz, C. FDA approves Gardasil 9 for more types of HPV. *Cancer* **121**, 1156-1157 (2015).

38. Ball, J.M.*, et al.* Recombinant Norwalk virus-like particles given orally to volunteers: phase I study. *Gastroenterology* **117**, 40-48 (1999).

39. Tacket, C.O.*, et al.* Human immune responses to a novel norwalk virus vaccine delivered in transgenic potatoes. *The Journal of infectious diseases* **182**, 302-305 (2000).

40. Kamili, S. Toward the development of a hepatitis E vaccine. *Virus research* **161**, 93-100 (2011).

41. Zhang, J.*, et al.* Long-term efficacy of a hepatitis E vaccine. *The New England journal of medicine* **372**, 914-922 (2015).

42. Wu, T.*, et al.* Hepatitis E vaccine development: a 14 year odyssey. *Human vaccines & immunotherapeutics* **8**, 823-827 (2012).

43. Proffitt, A. First HEV vaccine approved. *Nat Biotech* **30**, 300-300 (2012).

44. Weber, J.*, et al.* Immunogenicity of the yeast recombinant p17/p24:Ty virus-like particles (p24-VLP) in healthy volunteers. *Vaccine* **13**, 831-834 (1995).

45. Yusibov, V.*, et al.* Expression in plants and immunogenicity of plant virus-based experimental rabies vaccine. *Vaccine* **20**, 3155-3164 (2002).
